# Cup implantation accuracy using the HipCOMPASS mechanical intraoperative support device

**DOI:** 10.1186/s40064-016-2503-z

**Published:** 2016-06-18

**Authors:** Ken Suda, Tomoyuki Ito, Dai Miyasaka, Norio Imai, Izumi Minato, Naoto Endo

**Affiliations:** Department of Orthopedic Surgery, Niigata University, 757 1bancho, Chuoukuasahimatidori, Niigata City, Niigata 951-8510 Japan; Saiseikai Niigatadaini Hospital, 280-7, Nishikuteraji, Niigata City, Niigata 950-1104 Japan; Niigata Rinko Hospital, 114-3 1chome, Higashikumomoyamacho, Niigata City, Niigata 950-8725 Japan

## Abstract

**Background:**

While navigation systems have been developed to increase implantation accuracy in total hip arthroplasty (THA), they are not yet sufficiently versatile or commonly used. Therefore, to elevate the appeal of such systems, we have developed HipCOMPASS, a simple and effective mechanical angle indicator for use in supine THA.

**Questions/purposes:**

How accurate is the mean cup orientation [in terms of errors in radiographic anteversion (RA) and inclination (RI)] in cases where HipCOMPASS is used for intraoperative support? Does HipCOMPASS increase this cup orientation accuracy compared to THA cases without it? Does HipCOMPASS increase mean operation time?

**Methods:**

We measured cup orientation in 97 THA cases with HipCOMPASS and in 80 cases without it. Then we compared the angles determined in preoperative planning with the angles revealed by postoperative computed tomography (CT) for both groups. The discrepancy between them was defined as an error. Errors greater than 10° were considered outliers. Additionally, mean operative time with and without the Hip COMPASS were compared.

**Results:**

With the use of HipCOMPASS, the mean absolute error values in radiographic anteversion and inclination were 2.9° ± 2.3° (range 0°–12.8°) and 2.9° ± 2.1° (0.1°–7.7°), respectively. In contrast, without the use of HipCOMPASS, radiographic anteversion and inclination error values were 8.8° ± 5.8° (0.1°–25.4°) and 6.1° ± 4.5° (0.2°–21.0°), respectively. Outlier occurrence rates were 1.0 % with HipCOMPASS and 48.8 % without it. Mean operative times with and without HipCOMPASS use were 109.2 ± 23.8 min (74–199 min) and 137.6 ± 40.6 min (71–298 min), respectively.

**Conclusions:**

The study has found that HipCOMPASS dramatically increases implantation accuracy and it is also a simple and highly versatile tool that can be implemented quickly. Given its low cost in addition to its favourable accuracy, simple implementation, and short operative time, HipCOMPASS can be regarded as a very useful and effective THA support device.

**Level of evidence:**

Retrospective comparative study, Level 3.

## Introduction

### Background

Total hip arthroplasty (THA) has been greatly advanced by improvements in the quality of component materials and increased femoral head diameter. However, methods for enhancing the accuracy of acetabular cup implantation need be explored further to reduce risk of impingement, dislocation, imbalance, and wear (Lewinnek et al. 1978; Kennedy et al. 1998; Sanchez-Sotelo et al. 2006; Conroy et al. 2008; Patil et al. 2003; Widmer 2007; McCollum and Gray 1990; Sarmiento et al. 1990; Woo and Morrey 1982; Widmer and Zurfluh 2004; Daines and Dennis 2012; Beckmann et al. 2009).

While computer-assisted navigation can increase implantation accuracy (Jolles et al. 2004; Stiehl et al. 2007; Fukunishi et al. 2008; Nogler et al. 2004; Jenny et al. 2009; Broers and Jansing 2007), it is not widely used due to high cost, technical complexity, and extended operation time.

**Fig. 1:**
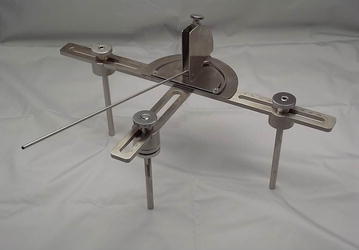
The HipCOMPASS mechanical intraoperative angle guide device. The device has legs to be placed on the *left* and *right* anterior superior iliac spines (ASIS) and on the pubic symphysis. The width to the *left* and *right* ASIS and the height to the pubic symphysis are variable, and adjusted to each individual patient. The length of the leg placed on the pubis is also adjustable, allowing for the correction of angle errors caused by soft tissue. The *left* and *right* ASIS legs are hollow, and the device is fixed to these ASIS by the insertion of Kirschner wires through them from above. An angle indicator is placed on the device, and the cup is inserted parallel to the direction of the indicator, thereby increasing its accuracy

Hence, it is necessary to develop a cup implantation guide device that might facilitate THA procedures in a more inexpensive way. To address this need, we developed and tested a device called HipCOMPASS, a mechanical intraoperative angle guide for use in supine THA (Fig. 1).

### Rationale

Initially, we formulated an idea based on a hypothesis that HipCOMPASS (1) attains acceptable accuracy and (2) does not significantly prolong operative time. In this paper, in order to prove this hypothesis, we discuss the impact of using HipCOMPASS in terms of (1) the accuracy of acetabular cup implantation [errors in radiographic anteversion (RA) and radiographic inclination (RI)], (2) the percentage of outliers (errors greater than 10°), and (3) operation time. This analysis was achieved by comparing the results of supine THA procedures with and without HipCOMPASS retrospectively.

### Study questions

How accurate is the mean cup orientation (in terms of errors in RA and RI) in cases where HipCOMPASS is used for intraoperative support?

Does HipCOMPASS increase the cup orientation accuracy compared to THA cases without it?

Does HipCOMPASS prolong mean operation time?

## Methods

### Study design and setting

This was a retrospective study. A total of two groups were compared: the first included cases that used HipCOMPASS (the “COMPASS group”), and the second included cases that did not use it (the “non-COMPASS group”). We started the study at the time that HipCOMPASS was introduced. The non-COMPASS group was analysed by comparing medical records and postoperative computed tomography (CT) data.

### Participants/study subjects

#### Subjects and THA procedures

The COMPASS group consisted of 97 patients [26 men, 71 women; mean age, 57.1 ± 9.1 years (range 37.3–77.5 years)] who underwent THA with HipCOMPASS between November 2010 and April 2012 at our institution. The non-COMPASS group consisted of 80 patients [14 men, 66 women; mean age, 59.2 ± 10.2 years (range 32.9–81.7 years)] who underwent THA before HipCOMPASS was introduced. A breakdown of the COMPASS group by preoperative Crowe Classification was as follows: type I: 64 patients (66.0 %); type II: 22 patients (22.7 %); type III: 10 patients (10.3 %); and type IV: one patient (1.0 %). The breakdown of the non-COMPASS group, on the other hand, was as follows: type I: 56 patients (70.0 %); type II: 15 patients (18.8 %); type III: eight patients (10.0 %); and type IV: one patient (1.3 %). Types I and II were defined as mild deformation, types III and IV were defined as advanced deformation, and the percentage of advanced deformation was 11.3 % in each group. No significant differences were observed between the groups in sex (*p* = 0.14), age (*p* = 0.22), or percentage of advanced deformation (*p* = 0.99) (Table 1).

**Table 1:** Patient characteristics

	COMPASS group	Non-COMPASS group	*p* value
Number of patients	97	80	
Sex	Males: 20 Females: 71	Males: 14 Females: 66	0.14
Age	57.1 ± 9.1 years	59.2 ± 10.2 years	0.22
Age range	37.3–77.5 years	32.9–81.7	
*Crowe Classification*			
Type I (n, %)	64 (66.0 %)	56 (70.0 %)	
Type II (n, %)	22 (22.7 %)	15 (18.8 %)	
Type III (n, %)	10 (10.3 %)	8 (10.0 %)	
Type IV (n, %)	1 (1.0 %)	1 (1.3 %)	
Number of advanced deformations (Type III + IV)	11 (11.3 %)	9 (11.3 %)	0.99

Prior to the use of HipCOMPASS, ethics board approval was obtained in our institution. All of the patients in the COMPASS group gave informed consent for treatment.

### Description of experiment, treatment, or surgery

The two groups were compared in terms of cup implantation angle error. Error was defined as the difference in angles determined during preoperative planning and those revealed postoperatively by CT. Plans were drafted with ZedHip three-dimensional preoperative planning software (Lexi, Tokyo, Japan). The horizontal plane of the CT table during preoperative CT in the supine position was defined as the functional pelvic plane (FPP), and the standard alignment of the acetabular component was defined as RA of 15° and RI of 45° relative to the FPP (Murray 1993). Operative times were calculated based on medical records and compared between the COMPASS and non-COMPASS groups. In both groups, the press-fit Plasmacup titanium shell (B. Braun Aesculap, Tuttlingen, Germany) and the Bicontact D cementless femoral stem (B. Braun Aesculap, Tuttlingen, Germany) were used. In all cases, THA was performed in the supine position with a minimally invasive surgical Watson-Jones approach, and the anterior hip capsule was removed. The surgeries were performed by two hip surgery specialists with several cases of experience and three orthopaedic surgeons with nearly 100 cases of experience.

#### The HipCOMPASS as an intraoperative device

**Fig. 2:**
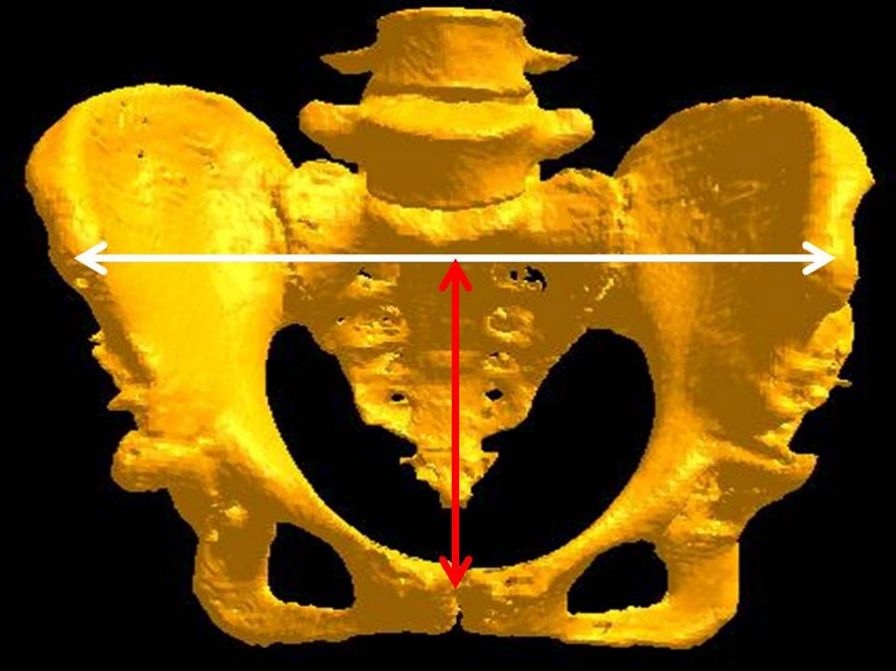
Based on a preoperative CT scan image, the length of the straight line connecting the *left* and *right* ASIS is defined as the pelvic width, while the distance between this line and the pubic symphysis midpoint is defined as the pelvic height

**Fig. 3:**
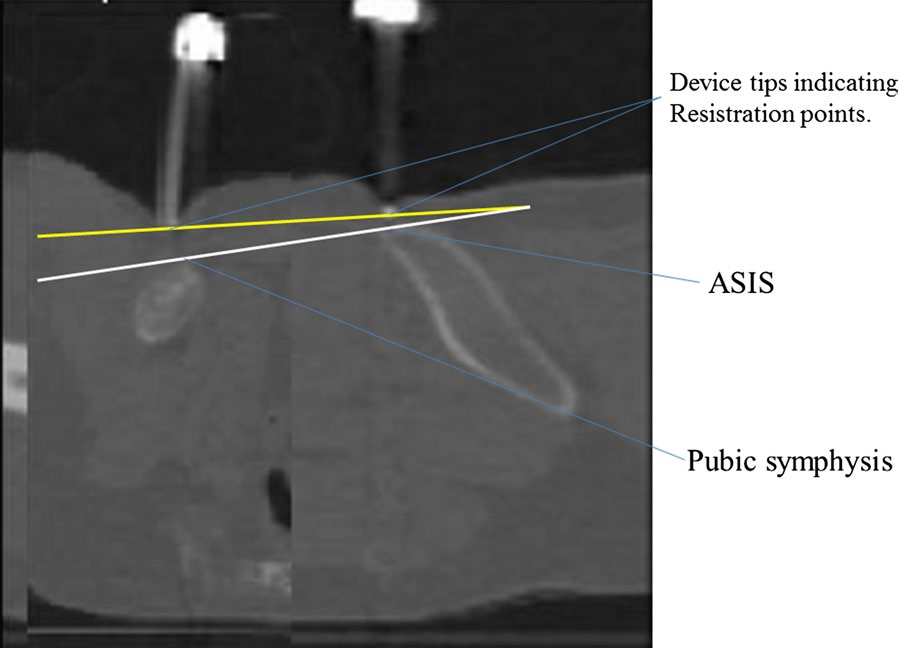
Composite schematic diagram on a sagittal CT section. The *yellow line* is the reproduced APP with the device tips in place, while the white line is the true APP. The angle formed by these *two lines* is the APP error caused by soft tissue, which requires correction

The left and right anterior superior iliac spines (ASIS) and the pubis are defined as the registration points, and the anterior pelvic plane (APP) formed by these three points is recreated in the anterior pelvis during THA. The body of HipCOMPASS has three attached legs that make contact with these three points. The width and height of HipCOMPASS are adjusted based on the width and height of the individual’s pelvis as measured during preoperative planning (Fig. 2). The device is placed on the skin and manipulated from the anterior portion; hence, it is prone to APP error due to soft tissue thickness (Fig. 3).

**Fig. 4:**
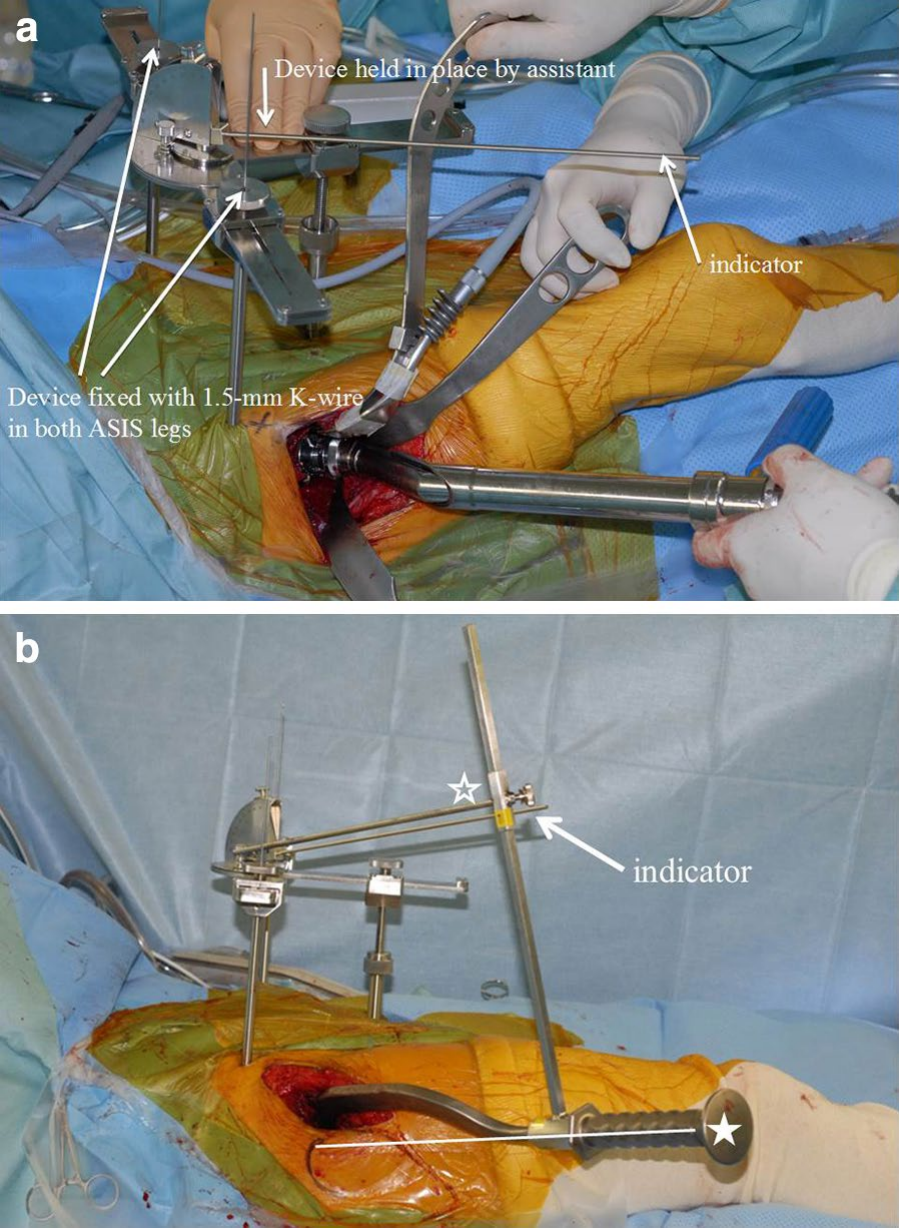
**a** After the device is adjusted for the pelvic width, pelvic height, and error caused by soft tissue thickness, the legs of HipCOMPASS are placed on the *left and right* ASIS and on the pubic symphysis. In this position, the device is fixed by insertion of 1.5-mm K-wires through the hollow *left and right* ASIS legs and into the ASIS. An assistant applies appropriate force to the anterior of HipCOMPASS to hold the device in place, thereby allowing the indicator to show accurate angles. **b** An additional device is attached to the side of the *cup* holder with a *bar* (*asterisk*) parallel to the orientation of the *cup* (*asterisks*), and the *cup* is inserted such that it sits parallel to the device indicator. The *bar* and the indicator are set very close to each other, allowing the *cup* to be inserted more accurately

To correct this error, we measured the thickness of the soft tissues above the left and right ASIS and the pubis in each patient by puncturing them with a special depth gauge under general anaesthesia just before THA. The tip of the gauge has the same shape and diameter as the tips of the legs of HipCOMPASS, which allows the thickness to be captured and measured by manipulation of the device. The mean thickness of the soft tissues above the left and right ASIS was subtracted from that of the pubis to correct sagittal rotation error. The difference in soft tissue thickness above the left and right ASIS was small in most patients, with a maximum thickness of approximately 2 mm. Considering the width of the pelvis, the axial rotation error was extremely small and was therefore ignored. The leg of the device that is placed on the pubis is the only leg that is adjustable. It can be shortened based on the calculated soft tissue thickness to equally match the inclines of the true APP and of the device body. The legs on the left and right ASIS were fixed by Kirschner wires with an internal diameter of 1.5 mm. An indicator was installed onto the device to recreate the same angles as determined in preoperative planning where the intraoperative cup alignment relative to the APP had automatically been calculated by ZedHip (Fig. 4a).

**Fig. 5:**
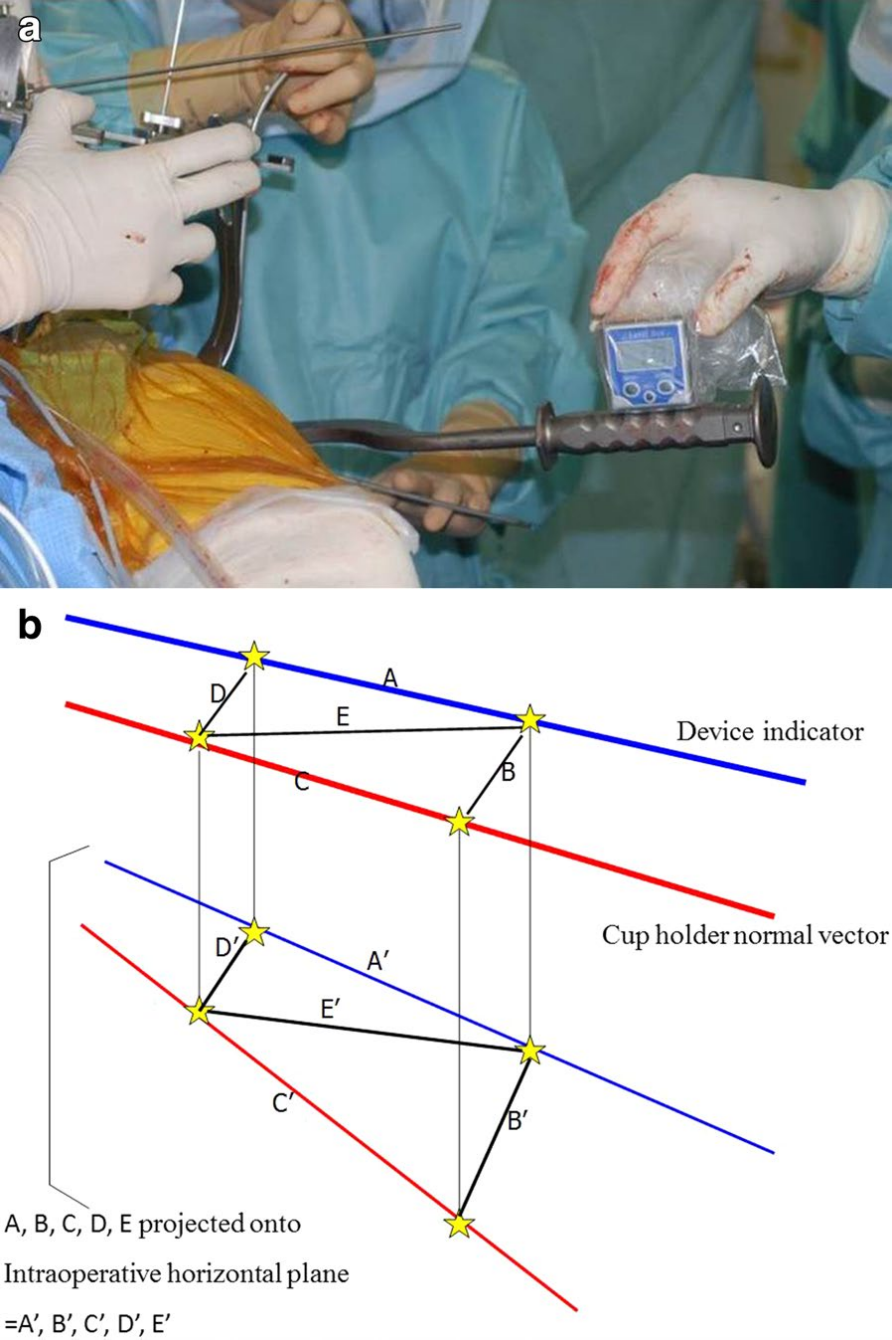
**a** To assess the accuracy of the angle of the indicator in RA, the bed is rotated and aligned so that the device itself is *horizontal*. At the same time, the inclines from the horizon of the cup holder and the indicator are each measured with a level gauge, and the difference between them is defined as the operator error in RA. **b** Similarly, to calculate the operator error in RI, two points are established on both the indicator and the *bar*, which is attached to and in parallel alignment with the cup holder, as shown in the diagram. The sides of the four-sided figure formed by these four points are defined as *A*, *B*, *C*, and *D*, while one of the diagonals is defined as *E*. *Lines* *A*–*E*, as projected onto the intraoperative horizontal plane, are defined as lines *A*ʹ–*E*ʹ and the lengths of each are calculated. Then, after calculation of the angle formed by *A*ʹ and *C*ʹ using the law of cosines, the angle formed by *C* as *A*ʹ is projected onto the plane where *C* lies is calculated, and defined as the operator error in RI

An additional bar parallel to the cup holder was attached to make the bar and the indicator closer, which improved the precision of the cup implantation (Fig. 4b). To assess the precision of the device’s indicator, the angle from the horizon of the indicator and the bar was measured with a level gauge, and the difference between them was defined as the operator error in RA (Fig. 5a).

Meanwhile, the following method was used to calculate the operator error in RI. Two points were marked on both the indicator and the bar, and the sides of the four-sided figure formed by these four points were defined as A, B, C and D, while one of the diagonals was defined as E. The lengths of these five lines were measured, and the angles from the intraoperative horizon were measured with a level gauge. By multiplying each of the lengths of lines A-E by the cosine of each of the angles with respect to the horizon, the lengths of lines A-E as projected onto the intraoperative horizontal plane were calculated as Aʹ, Bʹ, Cʹ, Dʹ, and Eʹ, respectively. We then calculated the angle formed by Aʹ and Cʹ using the law of cosines. Lastly, this angle, when moved in a coronal direction on the horizontal plane and projected onto the plane on which the normal vector of the cup holder lay, was calculated and defined as the operator error in RI (Fig. 5b).

### Variables, outcome measures, data sources, and bias

#### Accuracy assessment

A CT scan was performed within 2 weeks of THA for postoperative assessment. CT DICOM data were incorporated into ZedHip, and the cup implantation angles relative to the FPP and APP were automatically calculated by ZedHip for the postoperative evaluation. The difference in the implantation angles between preoperative planning and postoperative CT data was defined as implantation accuracy, and was calculated for both RA and RI. The previously described operator errors were subtracted, and the accuracy of the angles indicated by the device’s indicator was calculated.

### Statistical analysis, study size

Differences in number of outliers and mean operative time between the COMPASS and non-COMPASS groups were evaluated by independent t test.

### Other methods

#### Postoperative assessment accuracy examination

To examine the accuracy of the postoperative assessment itself, both inter- and intra-rater reliability were examined. The measurement values used in these examinations were the RA and RI of the cup. For inter-rater reliability, 10 randomly selected joints were measured once each by three raters. For intra-rater reliability, three randomly selected joints were measured 10 times each by a single rater. The intra-class correlation coefficient (ICC) was calculated for each item in order to ascertain reliability. Postoperative inter-rater reproducibility was good, with no major variance observed for any item. The intra-rater standard deviation was less than 1° for each item. Both the inter- and intra-rater ICC were considered excellent, at 0.98–0.99 for all items.

## Results

### How accurate is the mean cup orientation (in terms of errors in RA and RI) in cases where HipCOMPASS is used for intraoperative support?

In THA using HipCOMPASS, the mean absolute values of implantation errors in RA and RI were 2.9° ± 2.3° (range 0°–12.8°) and 2.9° ± 2.1° (range 0.1°–7.7°), respectively. The number of outliers (errors greater than 10°) was one joint out of 97 (1.0 %). Device indicator errors (subtracted by the operator errors) for RA and RI were 2.4° ± 2.2° (range 0–13.0°) and 2.5° ± 1.9° (range: 0–8.0°), respectively.

### Does HipCOMPASS increase cup orientation accuracy compared to THA cases without it?

**Fig. 6:**
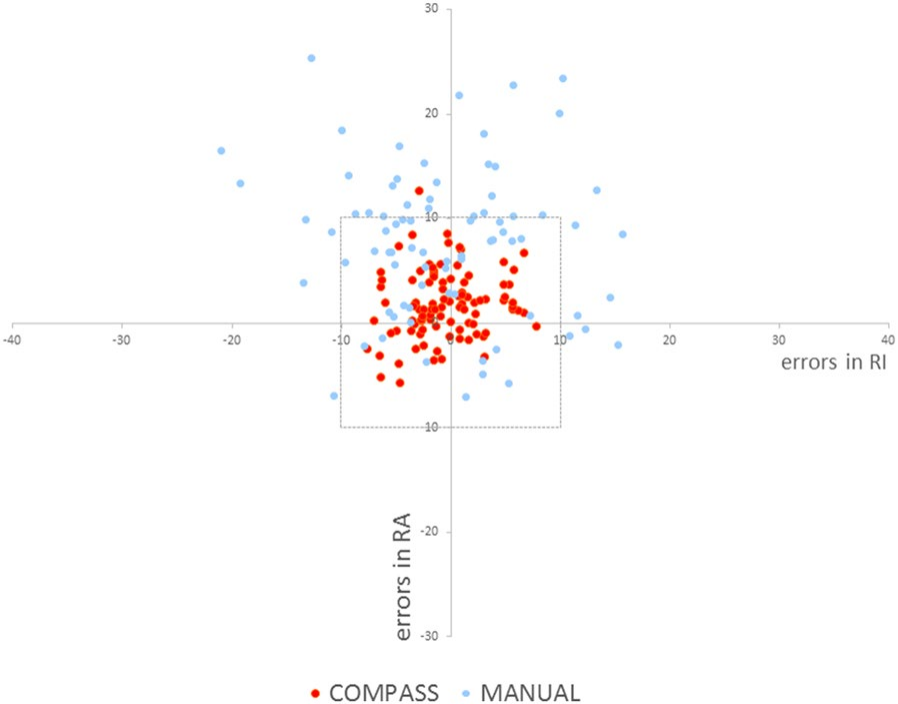
Cup implantation errors in the COMPASS and non-COMPASS groups are shown in a scatterplot. The COMPASS group contained one outlier (error greater than 10°) out of 97 patients (1.0 %), whereas the non-COMPASS group contained 39 outliers out of 80 patients (48.8 %); i.e. the COMPASS group contained significantly fewer outliers (*p* < 0.001)

In the non-COMPASS group, implantation errors in RA and RI were 8.8° ± 5.8° (range 0.1°–25.4°) and 6.1° ± 4.5° (range: 0.2–21.0°), respectively. The number of outliers was 39 joints out of 80 (48.8 %); thus, there were significantly fewer outliers in the COMPASS group (*p* < 0.001) (Fig. 6).

### Does HipCOMPASS prolong mean operation time?

Mean operative times in the COMPASS group and non-COMPASS groups were 109.2 ± 23.8 min (range 74–199 min) and 137.6 ± 40.6 min (range 71–298 min), respectively. Operative time was therefore significantly shorter in the COMPASS group (*p* < 0.001).

## Discussion

### Background and rationale

HipCOMPASS makes contact with the left and right ASIS and the pubic symphysis from the surface of the skin through the soft tissues. During THA, the APP is reproduced to serve as a reference for the implantation angle. The three registration points of the device are all fixed. Therefore, if two of them are placed accurately, the third should have correct placement as long as the size of the pelvis is measured precisely prior to THA. This configuration reduces the possibility of deviation of the registration points from the left and right ASIS and the pubis, thus reducing the likelihood of extremely large errors.

**Fig. 7:**
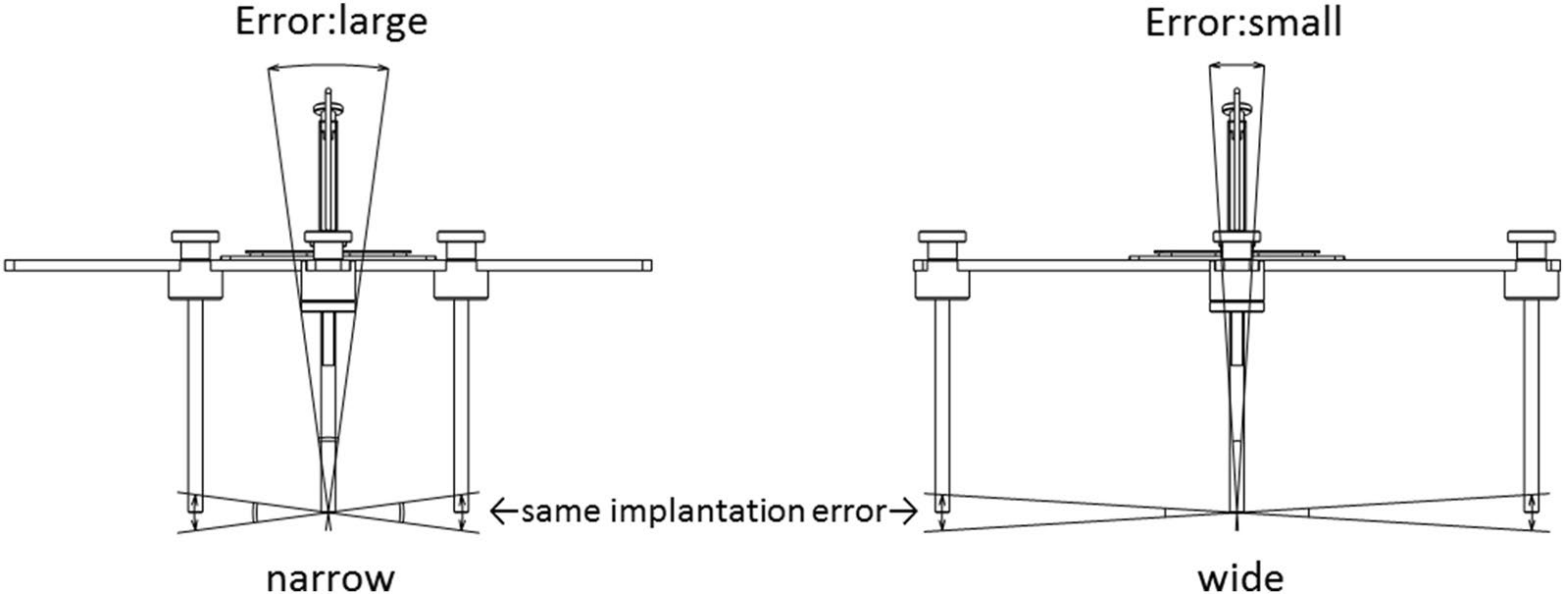
When implantation errors at the registration points are identical, the points with a greater width between them will have a smaller angle error. This can be said for all three-dimensional directions (*pitch*, *roll*, and *yaw*)

Any time that intraoperative contact is used during registration to determine the shape of the pelvis and as a reference for the cup implantation angle, a greater width of the registration points can reduce the angle error in all three-dimensional directions (pitch, roll, and yaw) if the error at each point is constant (Fig. 7). Since registration with HipCOMPASS is performed from the surface of the skin, the accuracy of each point is not as great as that of CT-based navigation. However, the left and right ASIS and the pubic symphysis provide the greatest width of all of the simple and usable points, which may be one reason why HipCOMPASS yielded a high level of implantation accuracy.

Steppacher et al. (2011) have reported favourable outcomes using the HipSextant intraoperative support device for THAs performed in the lateral position. Although the HipSextant is similar to our device in that registration is performed by obtaining three points on the pelvis, it differs from HipCOMPASS in many ways. Firstly, the HipSextant is placed directly on bone, which gives it greater accuracy at each registration point, and thus there is only a small possibility that the patient’s BMI will affect accuracy. With HipCOMPASS, registration is carried out on the surface of the skin; hence, although the accuracy of each point is inferior to that of the HipSextant, HipCOMPASS has the advantage of being minimally invasive. Furthermore, both devices are characterized by an ability to provide the maximum width of registration points, but HipCOMPASS is capable of providing a greater width. Additionally, while HipCOMPASS uses CT scan DICOM data due to its measurements of pelvic width and height, it does not require either a patient-specific 3D model or surface tracing, and is therefore easier to use.

Meanwhile, Zheng et al. (2011) developed PS-GANS: a patient-specific, gravity assisted navigation system for acetabular cup placement that has a reportedly high accuracy of cup orientation in lateral THA. Their excellent results may have been achieved by obtaining a greater width between two of the registration points, at the left and right ASIS. Although the device is indeed interesting, that report has several limitations. Firstly, their study is based on experiments using 3D shape model “dry bones” created by 2D–3D reconstruction, the accuracy of which is unknown. In an actual surgery, soft tissues can prevent accurate registration. Secondly, the authors do not consider costs, versatility, or prolongation of the operative time. Lastly, they define the orientation established by an image-free navigation system as the “ground truth”, but measurements obtained by such a system are not always accurate as the system may have errors in a small range (Jolles et al. 2004; Stiehl et al. 2007; Fukunishi et al. 2008; Nogler et al. 2004; Jenny et al. 2009; Broers and Jansing 2007).

The ultimate goal of enhancing implantation accuracy in THA is to reduce complications, such as dislocation and early wear. Thus, no matter how much intraoperative implantation accuracy values are improved, the likelihood of complications will not be decreased if the orientation and planning itself lacks validity. Various factors must be taken into consideration, such as the patient’s pelvic inclination in the standing, supine, and sitting positions; lumbar range of motion; and changes over time. It is important to have appropriate foresight and to improve implantation accuracy using intraoperative support from devices. However, many problems remained to be solved and there is still much room for discussion.

### Limitations

Likewise, HipCOMPASS itself also has limitations. To begin with, because it is employed at the surface of the skin, accurate implantation may not be possible for patients with extremely large BMI. Although we were able to perform implantation with the aid of HipCOMPASS in all 97 patients on whom we actually performed surgery, one of the volunteers at the device development stage had a BMI of 40, which made accurate implantation of the device difficult. In contrast, as mentioned above, HipSextant is placed directly on the bones, which gives it higher accuracy at each registration point and substantially limits the potential for the patient’s BMI to negatively affect accuracy (Steppacher et al. 2011). However, HipCOMPASS has the advantage of being minimally invasive by comparison. Another limitation is that the device is only an implantation angle indicator, and thus it cannot demonstrate the positional information that is included in CT-based navigation. Lastly, the device is only used for THA in the supine position at present. We are currently investigating applications of HipCOMPASS in THA in the lateral position.

#### *Discussion*

*How accurate is the mean cup orientation (in terms of errors in RA and RI) in cases where HipCOMPASS is used for intraoperative support?*

The results of mean cup orientation (errors in both RA and RI) were sufficiently small in the COMPASS group.

#### *Discussion*

*Does HipCOMPASS increase the accuracy of cup orientation compared to THA cases without it?*

In addition to the improvements in cup orientation with HipCOMPASS, there were many outliers in the non-COMPASS group, but only one outlier in the COMPASS group.

#### *Discussion*

*Does HipCOMPASS prolong mean operation time?*

The use of HipCOMPASS did not result in an increase in operation time. It is easy to apply once the operator develops the necessary skills, making it a very valuable device in supine THA.

## Conclusions

This study has shown that the supine THA application of HipCOMPASS, a mechanical intraoperative angle guide, dramatically increased cup implantation accuracy, reduced the rate of implantation errors greater than 10°, and did not prolong operative time. Given its low cost, along with its significantly high level of accuracy and minimal time requirement enabled by a simple means of implementation, it can be concluded that HipCOMPASS is a very useful tool in supine THA that can elevate the appeal of such navigation systems.
